# Supplementary material for: Muskrats are greater carriers of pathogenic Leptospira than coypus in ecosystems with temperate climates
Source: PLoS One. 2020 Feb 19;15(2):e0228577. doi: 10.1371/journal.pone.0228577 (PMC7029846; doi:10.1371/journal.pone.0228577)
Supplement: Attachment — Submitted filename: Response to reviewers.doc [file pone.0228577.s003.doc]

Marcy L’Étoile, Decembre 6, 2019

Dear Editor,

As suggested, we ensured that our manuscript met PLOS ONE's style requirements. We thus used Level 1 headings for all major sections (Abstract, Introduction, Materials and Methods, Results, Discussion, etc.), bold type, 18pt font. We modified the Figure citations and the figure captions according to the guidelines.

Considering your comment on [Figure(s) 2] that contains [map/satellite] images, which may be copyrighted, we fully understand the issue related to copyrights. The mapping in Figure 2 was generated using ArcGIS® and the background map: IGN GEOFLA®. We therefore cited the source as follows:

*“To assess potential variation in Leptospira serogroup distribution, the study area was divided into three administrative regions: Brittany, Normandy and Pays de la Loire. Data were visualized in ArcGIS version 9.3 (ESRI, Redland, CA, USA**) with the background map from IGN GEOFLA®.”*

The figure caption also includes “*Source background map: IGN GEOFLA*®.”

Please find below the point-by-point responses to each of the three reviewers’ comments.

**Reviewers #1 and #2:**

Is the manuscript presented in an intelligible fashion and written in standard English? Answers were “No”

To improve the flow of the paper, the phrasing and grammar, we have asked a native English speaker, Editor in the Life Sciences, to revise our manuscript. Required changes to grammar and style were made.

**Reviewers #1:**

Comments 1, 2, 4, 5, 7, 8, 9, 13 and 15 were related to typing errors or inappropriate phrasing. For each of his comment, the reviewer kindly gave some suggestions.

We agree with these remarks and modified the manuscript accordingly. We thank the reviewer for these suggestions which increase general understanding of the paper.

Comment 3: “*Author mentioned that “No animal” were killed. What does that mean? How was the kidney isolated for the study then? That is worrisome.”*

We agree that the ethics statement must be clarified to ensure clear understanding of the study context. We thus amended the sentence as follows:

*“****All samples were collected from rodents legally killed for population control; therefore, this study did not involve deliberate additional killing of animals and no ethical approval was considered necessary.*** *All procedures for population control complied with the ethical standards of the relevant national and European regulations on the care and use of animals (French authority Decision 2007/04/06 and Directive 2010/63/EC).”*

Comment 6*: “There were no information on how and what data was use for analysis. In the result section, there were percentage and confident interval used.”*

We agree with the reviewer’s comment. The information on how we obtained the prevalence, the seroprevalence and the confident intervals was missing; we therefore included the following sentence in the Materials and Methods section:

*“Apparent prevalence, seroprevalence, and 95% confidence intervals were calculated using exact binomial tests.”*

Comment 10: *“Wondering if the word infection in muskrat and infection in rat was appropriate. These animals are clinically healthy right? Rodents are known to be carrier and they just transmit the disease. “*

We thank the reviewer who has underlined that using ‘infection’ in rodents could be confusing or inappropriate. To clarify this point, we amended the sentence as follows:

*“Here, the results showed that the extent of Leptospira carriage* ***is similar in muskrat and rat populations.****”*

Comment 11: *« line 134. “cases” These cases refer to human cases or animal cases.”*

We agree that the “cases” needed to be clarified. We thus added “human cases” as the specificity stated was related to analysis in humans.

Comment 12:”*line 141. All the percentages. What do they mean. Which one is for human and which percentage is for the livestock? Which type of livestock? Need clarity or detail*.”

We agree that this sentence should be clarified to improve the flow of the paper and for a better understanding of the reader. We amended the sentence as follows:,

*“Based on the high renal carriage and the pathogenic strain of Leptospira found in muskrats, which was the same strain found in patients kayaking in the same region some years later, but also in* ***a number of leptospirosis cases in humans (3% to 24%) and cattle (17%) in France****...”*

Comment 14: “*line 155. The present study confirms…How did this study confirm the infection risk. There were no human being samples. You are merely referring to the other study and both studies had occurred on two different time point*.”

We agree that “confirm” was not appropriate. We modified the sentence as follows:

*“[…] the presence of muskrats* ***appears to be*** *a considerable risk factor for humans and domestic animals.”*

Comment 16 and 17: One sentence as a stand-alone paragraph. Please check.

Where is the conclusion?

We agree that a conclusion could improve the flow of the paper and we followed the reviewer suggestion. We thus included the following conclusion,

*“This study shows that muskrats and coypus are important carriers of pathogenic Leptospira in aquatic environments in temperate climates. The serogroups Australis and Grippotyphosa were found to be predominant in coypus and muskrats, respectively, and the highest prevalence was observed in muskrats. Like coypus, muskrats are an invasive species of semi-aquatic rodents and their abundance can be high in water bodies possibly frequented by people and domestic animals. The presence of this species should be considered a risk factor for human and domestic animal leptospirosis and taken into account by public health policy makers, especially in terms of prevention and population control.”*
