# Supplementary material for: Muskrats are greater carriers of pathogenic Leptospira than coypus in ecosystems with temperate climates
Source: PLoS One. 2020 Feb 19;15(2):e0228577. doi: 10.1371/journal.pone.0228577 (PMC7029846; doi:10.1371/journal.pone.0228577)
Supplement: Attachment — Submitted filename: Reviewer comment for Plos.pdf [file pone.0228577.s002.pdf]

REVIEWER COMMENTS

"MUSKRATS ARE GREATER CARRIERS OF PATHOGENIC LEPTOSPIRA THAN COYPUS IN ECOSYSTEMS WITH TEMPERATE CLIMATES"

1. Line 42. ……habitat, with a resulting…… Suggest to delete with a

2. Line 51. The word "individual" was used by author but my guess that it referred to the rodent. Individual are for people/human. Please reword. Same in line 63, 92, 101. Please indicate properly, was it the rodents, the muskrats or the coypus.

3. Line 51. Author mentioned that "no animal" were killed. What does that means? How was the kidney isolated for the study then? That is worrisome.

4. Line 68. Spell out Ct in full. You are using the abbreviation for the first time.

5. Line 73. copenhagani. Use capital C.

6. After line 89. There were no information on how and what data was use for analysis. In the result section, there were percentage and confident interval used.

7. Line 96. Suggest that "regardless of the titer cut-off". I think that is understood and the lowest cut of point was 1:100 and the dilution method you use cannot go lower than that. That statement is redundant. Same in Line 109.

8. Line 99. Suggest use seroreaction instead of seroreacted.

9. Line 120. Please avoid using "we". All the "we" used in the entire manuscript was deemed inappropriate. Please change.

10. Line 129. Pondering if the word infection in muskrat ………and infection in rat was approriate. This animal are clinically healthy right? Rodents are known to be carrier and they just transmit the disease.

11. Line 134. "cases". These cases refer to human cases or animal cases.

12. Line 141. all the percentages. What do they mean? Which one is for human and which percentage is for the livestock? Which type of livestock? Need clarity or detail.

13. Line 147. Suggest "almost all results obtained" to "majority of the coypus (84%) were predominantly identified with ………."

14. Line 155. The present study confirm……….how did this study confirm the infection risk. There were no human being samples. You are merely referring to the other study and both study had occurred on two different time point.

15. Line 160. Strain. Was it serogroup?

16. Line 163. Redo. One sentence as a stand-alone paragraph. Please check.

17. After line 166. Where is the conclusion?
